# Diagnosing asymptomatic tuberculosis: current approaches, emerging tools, and persistent challenges

**DOI:** 10.3389/fcimb.2026.1935260

**Published:** 2026-10-27

**Authors:** Mao-Shui Wang, Dong-Yan Wei, Xu-Hui Liu, Zhong-Fa Zhang

**Affiliations:** 1 Department of Lab Medicine, Shandong Public Health Clinical Center, Shandong University, Jinan, China; 2 Department Pediatrics, Affiliated Hospital of Youjiang Medical University for Nationalities, Baise, Guangxi, China; 3 Department Pediatrics, The People’s Hospital of Chongzuo, Chongzuo, Guangxi, China; 4 Department of Tuberculosis, Shanghai Public Health Clinical Center, Fudan University, Shanghai, China; 5 Department of Respiratory Medicine, Shandong Public Health Clinical Center, Shandong University, Jinan, China

**Keywords:** active case finding, asymptomatic tuberculosis, chest radiography, computer-aided detection, diagnosis, host biomarkers, subclinical tuberculosis

## Abstract

Asymptomatic tuberculosis is a major but incompletely defined manifestation of the tuberculosis disease spectrum and an important challenge for active case finding. It is best understood as a symptom-defined and method-dependent phenotype rather than a uniform biological stage. Individuals classified as asymptomatic may have minimal or extensive radiographic abnormalities, low or intermittent bacillary shedding, or clinically inapparent disease that remains stable, regresses, fluctuates, or progresses. Differences in terminology, symptom ascertainment, population risk and diagnostic intensity have therefore led to substantial variation in how asymptomatic or subclinical tuberculosis is recognized and interpreted across studies and programmes. This Review examines current and emerging approaches for diagnosing asymptomatic tuberculosis, with emphasis on the interaction between epidemiological risk, radiological detectability, microbiological confirmation and longitudinal follow-up. Symptom-based screening and passive case finding miss a substantial proportion of bacteriologically or radiographically evident tuberculosis. Conventional sputum-based testing is constrained by difficulty obtaining specimens, paucibacillary disease, intermittent shedding and imperfect sensitivity. Chest radiography and computer-aided detection can expand screening capacity and standardize triage, but they cannot independently establish disease activity or exclude tuberculosis. Computed tomography and positron emission tomography/computed tomography provide more sensitive characterization in selected settings, although their cost, accessibility and limited specificity preclude routine use. Non-sputum pathogen assays and host biomarkers are promising because they may reduce dependence on spontaneous sputum, but most remain insufficiently validated, and their incremental clinical or programmatic value over imaging-based triage and conventional confirmation remains uncertain. The diagnostic challenge of asymptomatic tuberculosis lies not simply in the absence of symptoms, but in the unstable boundary between infection, early disease, radiological abnormality, microbiological detectability and future risk. Clinical and public health practice should therefore move beyond single-test strategies toward staged, risk-stratified diagnostic pathways that integrate symptom ascertainment, exposure risk, imaging, confirmatory microbiology, enhanced specimen collection and longitudinal reassessment. Such pathways should be viewed as flexible clinical-programmatic frameworks for organizing diagnostic uncertainty, guiding multidisciplinary decisions and generating testable hypotheses, rather than as validated treatment algorithms.

## Introduction

Asymptomatic tuberculosis (TB) is a symptom-defined phenotype rather than a uniform biological state (Coussens et al., 2024; Teo et al., 2024; Dheda et al., 2025). This distinction is important because TB is increasingly conceptualized as a dynamic disease spectrum rather than a binary latent–active state, encompassing *Mycobacterium tuberculosis* infection and a range of incipient, subclinical and symptomatic disease states (Richards et al., 2023; Coussens et al., 2024). Asymptomatic TB is biologically and clinically heterogeneous. Some individuals have minimal radiographic abnormalities or low-level bacillary shedding, whereas others have extensive lesions, cavitation, or a substantial microbiological burden. Disease may remain stable, regress spontaneously, fluctuate between detectable and undetectable states, or progress to symptomatic TB (**Figure 1**) (Richards et al., 2023). It should therefore not be assumed to represent uniformly early, paucibacillary, or clinically inconsequential disease.

**Figure 1:**
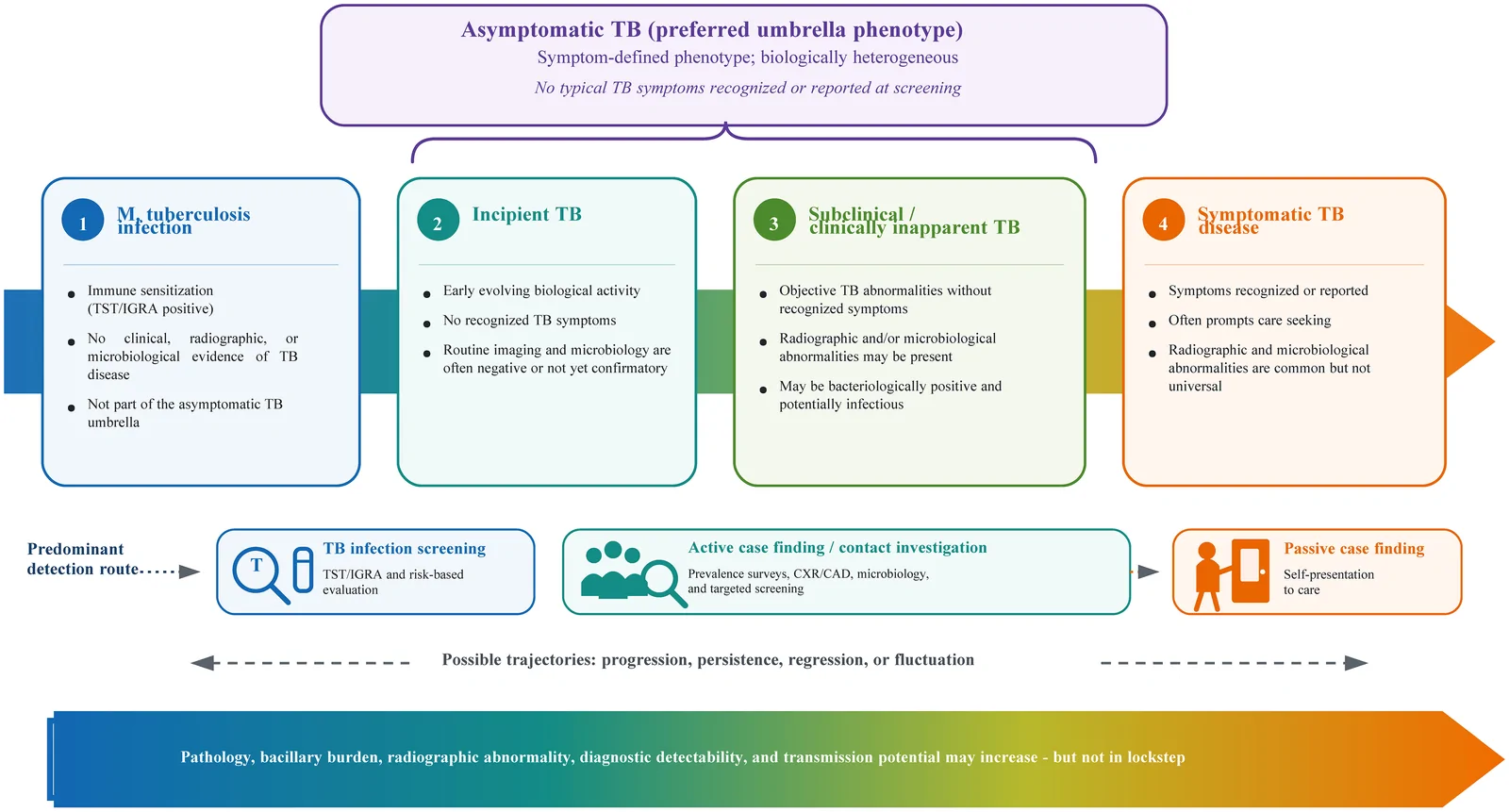
Asymptomatic tuberculosis across the tuberculosis disease spectrum. In this schematic, asymptomatic TB is used as a symptom-defined umbrella phenotype to denote individuals in whom typical TB symptoms are not recognized or reported at screening, rather than as a discrete disease-state category. Incipient TB is depicted to represent an early, evolving trajectory that may remain symptom-negative; consistent with the 2024 ICE-TB consensus, it should not be interpreted as a distinct, fixed disease state. Subclinical or clinically inapparent TB represents TB disease with objective abnormalities but without recognized symptoms. These phenotypes and trajectories are biologically heterogeneous, and individuals may progress, persist, regress, or fluctuate over time. Movement across the TB spectrum is therefore dynamic and not necessarily unidirectional.

Identifying asymptomatic TB is important because symptom-based screening fails to identify some individuals with microbiological or radiographic evidence of disease (Stuck et al., 2024; Mendelsohn et al., 2025). Asymptomatic disease may also contribute to transmission. *M. tuberculosis*-containing aerosols can be released during natural respiratory activity in the absence of recorded cough (Chen et al., 2026), although the contribution of specific non-cough respiratory maneuvers to viable bacillary transmission remains uncertain (Patterson and Wood, 2019). Genomic reconstruction suggests that some transmission can occur before recognized symptom onset, whereas population-level modeling indicates that subclinical TB may contribute substantially to overall transmission; however, these estimates are highly uncertain and are not directly comparable (Xu et al., 2019; Emery et al., 2023). Transmission potential is unlikely to be uniform across asymptomatic individuals. At the population level, the contribution of asymptomatic disease may be amplified by its high prevalence, potentially prolonged duration, and bacillary shedding in some individuals, although direct transmission from strictly asymptomatic cases remains poorly quantified (Kendall et al., 2021; Emery et al., 2023).

Diagnosis remains challenging because symptom-negative individuals often cannot produce spontaneous sputum and may have low, intermittent, or anatomically compartmentalized bacillary burdens (Lewinsohn et al., 2017; Mendelsohn et al., 2025; Shi et al., 2025). This Review addresses asymptomatic TB in both adults and children. It aims to clarify how definitions influence case classification; evaluate the strengths and limitations of conventional and emerging diagnostic approaches; highlight age-specific differences in symptom assessment, specimen collection, microbiological confirmation and imaging; and consider how host biomarkers, computer-aided imaging and artificial intelligence (AI) could be incorporated into risk-stratified, multimodal diagnostic pathways (**Figure 2**). For clinical and epidemiological evidence, we use the WHO operational definition of asymptomatic TB as TB disease in a person who does not report symptoms suggestive of TB during screening, further classified as bacteriologically confirmed or bacteriologically unconfirmed (WHO, 2025). In the conceptual disease-spectrum framework shown in **Figure 1**, however, “asymptomatic” is also used descriptively as a symptom-defined phenotype across symptom-negative trajectories and disease states; this broader schematic usage should not be interpreted as a formal disease-state classification. Accordingly, “asymptomatic TB” is used as the primary term throughout, whereas “subclinical TB” is retained when referring to studies that used this terminology (Dheda et al., 2025).

**Figure 2:**
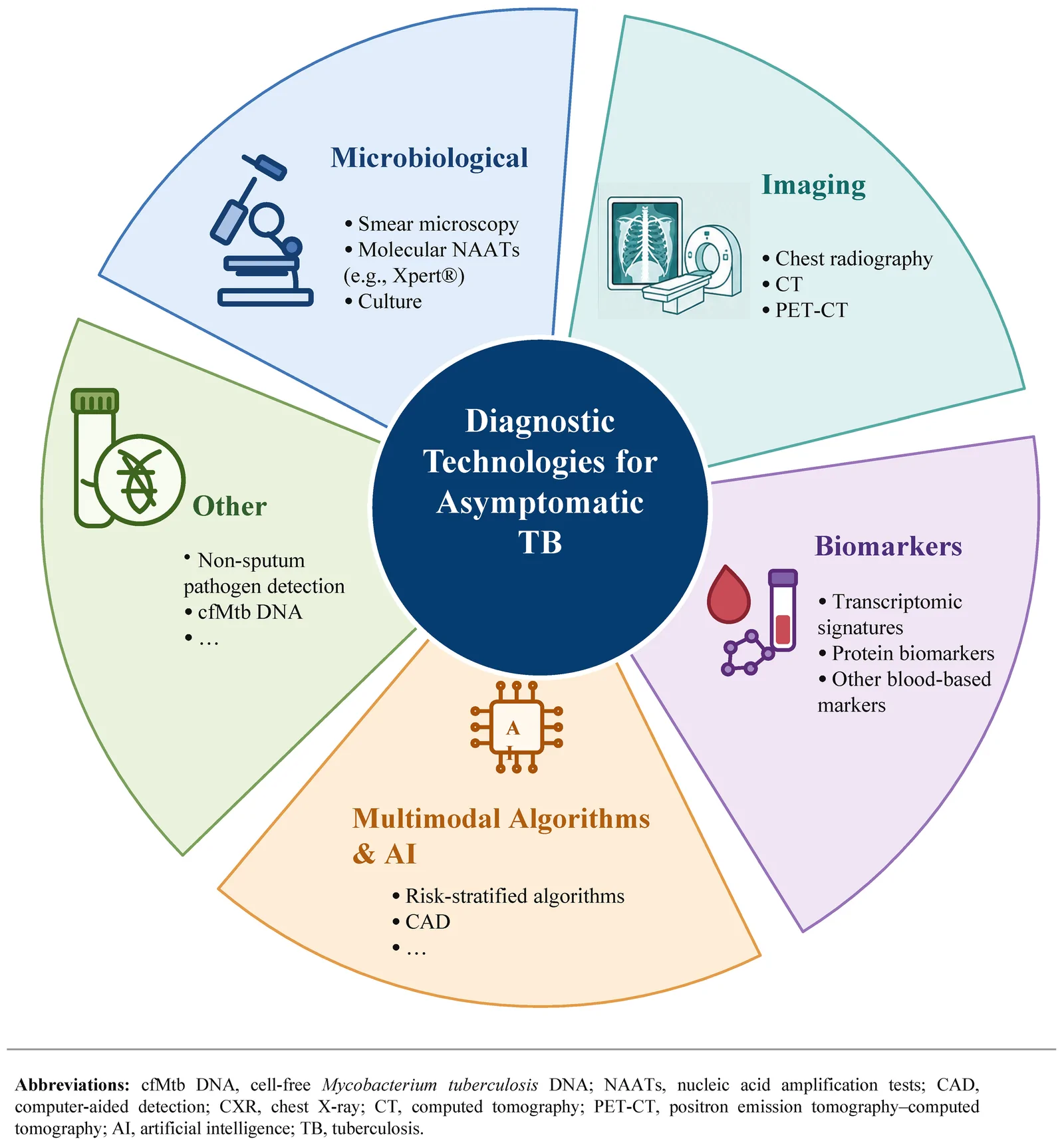
Current and emerging diagnostic technologies for asymptomatic tuberculosis.

This narrative Review was informed by a targeted, non-systematic search of PubMed rather than by a systematic review or meta-analysis. PubMed was searched from database inception to 25 May 2026 using combinations of the terms “subclinical”, “asymptomatic” and “tuberculosis”. The search was designed to identify relevant evidence on definitions, epidemiological burden, diagnostic performance and yield, clinical utility, and implementation of diagnostic approaches for asymptomatic TB. Studies were selected for discussion according to their relevance to the diagnostic questions addressed in each section, with particular emphasis on evidence directly evaluating asymptomatic or subclinical TB. Because study identification and selection did not follow a formal systematic-review protocol, no record-level screening or exclusion log was generated and no PRISMA-style flow diagram was constructed. This Review should therefore be interpreted as a structured narrative synthesis rather than an exhaustive or quantitatively comprehensive assessment of the available evidence.

## Epidemiological burden

TB control still relies heavily on symptom screening and passive case finding, which preferentially detect individuals whose symptoms prompt care seeking. Yet prevalence surveys and active case-finding studies show that a substantial proportion of individuals with bacteriologically confirmed pulmonary TB report no symptoms at screening. Estimates of this burden depend critically on the denominator used: prevalence refers to asymptomatic TB among the population screened, whereas proportion refers to the fraction of diagnosed TB cases that are asymptomatic. Across published studies, approximately 27–82% of prevalent TB has been classified as asymptomatic, depending largely on the symptom definition applied (Zaidi et al., 2026). In a recent systematic review and meta-analysis of adolescents and young adults aged 10–24 years, the pooled prevalence of asymptomatic TB among those screened was 1% (95% CI, 1–3%), whereas 62% (95% CI, 40–80%) of diagnosed TB cases were asymptomatic (Heidari et al., 2026). Similarly, an individual-participant-data meta-analysis of community-based prevalence surveys in adults found that the adjusted proportions of pulmonary TB without persistent cough, without any cough and without any reported TB-related symptoms were 82.8%, 62.5% and 27.7%, respectively (Stuck et al., 2024). Together, these findings highlight both the substantial burden of asymptomatic TB and the extent to which estimates vary according to population, case-finding strategy and symptom definition. Symptom screening also failed to identify most radiographically suspected cases among pregnant women with human immunodeficiency virus (HIV) in western Kenya (Kosgei et al., 2011).

Because asymptomatic TB rarely prompts spontaneous presentation, it is detected mainly through prevalence surveys, community screening, contact investigation and screening of high-risk populations (Dheda et al., 2025; Zaidi et al., 2026). Burden estimates therefore depend on the population screened, symptom definitions, diagnostic methods and intensity of case finding (Stuck et al., 2024; Teo et al., 2024; Chew et al., 2025; Zaidi et al., 2026). In a six-year Swiss surveillance study, subclinical TB accounted for 43 of 138 pediatric TB cases (31%); among children with a documented reason for investigation, 80.6% were assessed because of contact exposure (Fritschi et al., 2022). These findings illustrate how symptom-based surveillance can underestimate clinically inapparent disease.

Symptom ascertainment itself is also vulnerable to reporting bias. In a Vietnamese active case-finding study, 64.8% of bacteriologically confirmed cases reported no respiratory symptoms before diagnosis, compared with 17.8% after diagnostic disclosure (κ = 0.14), despite some having smear grade 1+, medium Xpert results, or TB-consistent chest radiographs (Teo et al., 2025).

Quantification is further complicated by inconsistent terminology and symptom thresholds. Historically, asymptomatic or subclinical TB has most commonly been defined by the complete absence of TB-related symptoms, the absence of cough, or the absence of symptoms exceeding prespecified duration or severity thresholds. In a review of 71 studies, only 27 provided an explicit definition. The estimated proportion was 45% (95% confidence interval [CI] 35–56%) when defined by the absence of any symptoms and 60% (55–65%) when defined by the absence of cough (Teo et al., 2024). Similarly, in a Singapore registry of 18,693 individuals with culture-confirmed pulmonary TB, prevalence was 41.6% when the definition included no cough or cough lasting less than 2 weeks, but 31.6% when restricted to complete absence of cough (Chew et al., 2025). Thus, the apparent burden depends strongly on how symptoms are defined and ascertained.

## Diagnostic challenges

Reference standards varied considerably across the studies reviewed. Asymptomatic status was generally defined by the absence of prespecified TB-related symptoms at screening, whereas confirmation of TB disease relied on study-specific diagnostic criteria. In adult screening and diagnostic-accuracy studies, microbiological confirmation by sputum nucleic acid amplification testing and/or mycobacterial culture was the predominant reference standard. By contrast, some pediatric and operational studies used composite microbiological, radiographic and clinical criteria, while longitudinal studies occasionally incorporated subsequent microbiological or clinical confirmation during follow-up. These methodological differences, together with heterogeneity in symptom definitions, diagnostic thresholds, study populations and age composition, limit direct comparison of diagnostic-performance estimates across studies and modalities.

### Age-specific considerations in adults and children

Diagnostic challenges differ substantially between adults and children with asymptomatic TB. In children, symptom assessment may be particularly difficult because symptoms are often mild or nonspecific, especially in younger age groups, and many children are unable to expectorate sputum. Respiratory sampling may therefore require induced sputum, gastric aspiration or nasopharyngeal aspiration (Lewinsohn et al., 2017). Consequently, microbiological confirmation is less frequently achieved; in a Swiss national surveillance study, only 34.9% of pediatric subclinical TB cases were microbiologically confirmed (Fritschi et al., 2022). Diagnosis in children therefore often depends more heavily on integrating exposure history, immunological testing, imaging and microbiological testing where feasible.

Imaging findings also require age-specific interpretation. In asymptomatic children, isolated hilar lymphadenopathy may be difficult to interpret (Gwee et al., 2013), and inter-observer agreement for hilar lymphadenopathy on chest radiography has been poor (Triasih et al., 2015). Among exposed children, radiographic abnormalities compatible with TB may be common, but such findings should not be interpreted as independent estimates of imaging sensitivity when radiographic features contribute to the case definition (Mulenga et al., 2026). By contrast, much of the evidence summarized in this Review on CT characterization (Lau et al., 2022), blood-based host signatures (Sivakumaran et al., 2023), CAD-assisted screening (Macpherson et al., 2025; Scott et al., 2025), and PET/CT (Esmail et al., 2026) derives from adult populations. Diagnostic performance estimates from adult cohorts should therefore not be assumed to apply directly to children, and pediatric-specific validation remains an important research priority.

### Limited sensitivity of pathogen-based testing

People with asymptomatic TB often do not cough and may be unable to expectorate, limiting spontaneous sputum collection. Obtaining respiratory specimens can be challenging in individuals who are unable to expectorate spontaneously. In children, specimen collection may require induced sputum, gastric aspiration, or nasopharyngeal aspiration, which involve additional procedures, trained personnel, and patient cooperation; adults who cannot expectorate may similarly require sputum induction or more invasive respiratory sampling (Lewinsohn et al., 2017). Specimen scarcity reduces completion of pathogen-based testing and increases the risk of missed disease (Lewinsohn et al., 2017; Mendelsohn et al., 2025). Non-sputum sampling is promising, but current approaches remain incompletely validated or insufficiently sensitive for routine screening (Tschan et al., 2024; Daniel et al., 2026; Wood et al., 2026). In South Africa, using positivity by sputum Xpert Ultra or liquid culture as the microbiological reference standard, tongue-swab high-volume quantitative polymerase chain reaction (PCR) assay had a sensitivity of 35.1% for asymptomatic TB. Sensitivity declined markedly with bacillary burden: positivity was 100% among asymptomatic participants with high or medium Xpert Ultra grades, but only 19% among those with low, very low or trace grades (Wood et al., 2026). In a Tanzanian case–control study, with sputum Xpert MTB/RIF or Xpert Ultra used as the contemporaneous microbiological comparator, a sequence-specific hybridization assay for detecting *M. tuberculosis* cell-free DNA in urine yielded positive results in four asymptomatic household contacts (Tschan et al., 2024). Two were concurrently positive by sputum Xpert, whereas the other two were negative by both sputum Xpert and culture at enrolment but subsequently developed active TB.

Asymptomatic TB is often paucibacillary, resulting in lower diagnostic yields from smear microscopy, culture and molecular assays. Negative results therefore do not exclude disease, particularly in individuals with radiographic abnormalities or substantial epidemiological risk. Studies from South Korea and China consistently reported lower microbiological test positivity in subclinical than in symptomatic TB (Min et al., 2020; Tang et al., 2022; Jeong et al., 2023; Shi et al., 2025). In a nationwide Korean cohort, positivity rates for acid-fast bacilli smear microscopy, mycobacterial culture and TB PCR were 16.2%, 50.2% and 33.8%, respectively, in subclinical TB, compared with 35.9%, 66.3% and 56.9% in symptomatic TB (Jeong et al., 2023). Similarly, a Chinese multicenter study reported positivity rates of 11.5%, 40.6% and 40.6% for smear microscopy, culture and Xpert, respectively, in subclinical TB, compared with 25.2%, 59.2% and 63.7% in symptomatic disease (Shi et al., 2025). An Italian multicenter cohort showed the same pattern, with lower microscopy and culture positivity in asymptomatic than in symptomatic TB (58.7% versus 81.4% and 82.5% versus 93.7%, respectively) (Cotugno et al., 2025). However, this pattern is not universal. Among untreated South African people living with HIV, smear positivity was numerically higher in subclinical than in symptomatic TB (29% versus 14%), although the difference was not statistically significant (P = 0.08) (Bajema et al., 2019).

Combining diagnostic modalities can partly compensate for the limited sensitivity of individual tests. In a Swiss pediatric series, only 34.9% of subclinical cases were microbiologically confirmed, supporting an approach that combines immunodiagnostic testing and imaging with subsequent culture and molecular testing (Fritschi et al., 2022). Diagnostic yield can also be increased by changing the testing strategy. In South African household contacts, 82.4% of confirmed cases were asymptomatic and only 31.0% were smear-positive. Universal sputum testing identified 18 asymptomatic cases that would have been missed by symptom- or chest-radiography-triggered investigation, increasing diagnostic yield by 54.5% (Mendelsohn et al., 2025).

Culture provides important incremental yield, particularly in vulnerable populations, but is slow and incompletely sensitive. Among pregnant women with HIV in Soweto, all seven culture-positive cases had negative World Health Organization (WHO) symptom screens, 86% were smear-negative and the median time to culture positivity was 17 days (Rickman et al., 2020). Recurrent subclinical TB among people living with HIV receiving antiretroviral therapy was also predominantly culture-dependent: 82.3% of cases were smear-negative but culture-positive (Naidoo et al., 2022). Cohorts in Tanzania and Cape Town similarly showed that culture detected cases missed by symptom screening and radiography; however, delayed confirmation led some patients to be misclassified as having latent TB infection and to receive inappropriate isoniazid monotherapy (Mtei et al., 2005; Oni et al., 2011).

Pathogen-specific non-sputum assays may help overcome these limitations. A droplet digital PCR assay targeting IS6110 and IS1081 detected circulating M. tuberculosis cell-free DNA in 10 of 11 subclinical pulmonary TB cases (sensitivity, 90.9%); in that study, asymptomatic TB was classified on the basis of positive Xpert MTB/RIF or, when Xpert and culture were unavailable or negative, positive AFB smear microscopy (Daniel et al., 2026). However, these findings remain proof-of-concept because they were derived from stored plasma and small subgroups (Daniel et al., 2026). Pathogen-derived circulating proteins may offer greater biological specificity, but the evidence remains preliminary. Optimized mass spectrometry detected 19 *M. tuberculosis* proteins in serum-derived extracellular vesicles, including differential abundance of Rv2997, HspX, GroEL2, GroES and an MtrB peptide in early or asymptomatic TB. However, the study did not establish diagnostic thresholds or report sensitivity and specificity, and the findings have not undergone external validation (Nieto Ramirez et al., 2026).

### Value and limitations of radiographic screening

Chest radiography (CXR) can reveal abnormalities suggestive of TB in symptom-negative individuals, but such findings require cautious interpretation. Among asymptomatic children with a positive tuberculin skin test and/or interferon-γ release assay, routine CXR identified isolated non-calcified hilar lymphadenopathy in 6 of 26 children classified as having asymptomatic TB, although the diagnostic significance of this finding remained ambiguous (Gwee et al., 2013). In another cohort of children with household TB exposure, CXR was compatible with TB in 30 of 41 (73.2%) asymptomatic children who underwent imaging (Mulenga et al., 2026). However, this proportion cannot be interpreted as an independent estimate of CXR sensitivity because the asymptomatic TB group included unconfirmed cases for whom a compatible CXR contributed directly to the diagnostic classification (Mulenga et al., 2026).

Conversely, a normal CXR does not exclude active TB. Among asymptomatic adults with HIV and TB in Cape Town, 10 of 14 who underwent CXR had normal findings (Oni et al., 2011). In a Canadian cohort of culture-positive subclinical pulmonary TB, 17.5% had a normal CXR and 86.4% had minimal or no pulmonary parenchymal abnormality (Lau et al., 2022). Computed tomography (CT) was substantially more sensitive than CXR for cavitation (27.7% versus 7.4%), endobronchial spread (59.6% versus 7.4%), intrathoracic lymph-node enlargement (22.3% versus 5.3%), bilateral disease (38.3% versus 23.4%), and moderate-to-far-advanced or miliary parenchymal disease (42.6% versus 19.2%) (Lau et al., 2022). Among 14 patients without parenchymal abnormalities on CXR, CT identified minimal disease in seven and moderately advanced cavitary disease in one (Lau et al., 2022).

Interpretation is further limited by poor reproducibility and imperfect specificity. Among Indonesian child contacts, inter-observer agreement for hilar lymphadenopathy was poor (κ = −0.03 to 0.25), and agreement remained limited even for classifying radiographs as normal (Triasih et al., 2015). In the Canadian cohort, field readers missed cavitation in 68.0–81.8% of radiographs classified as cavitary by the respective expert comparator. They were also less likely than expert consensus to identify parenchymal abnormalities or a typical adult-type TB pattern; nevertheless, expert consensus classified 18.7% of cases as having no parenchymal abnormality (Long et al., 2023). Imaging may also misdirect evaluation toward malignancy. In a highly selected cohort of 22 asymptomatic patients with pulmonary TB initially suspected to have lung cancer, CT showed spiculated margins in 81.8% and lobulation in 68.2%. Among the nine patients who underwent fluorine-18 fluorodeoxyglucose positron emission tomography/computed tomography (^18^F-FDG PET/CT), all lesions had a maximum standardized uptake value (SUV_max_) above 2.5. Although mean SUV_max_ was lower in TB than in lung cancer, discrimination was incomplete (area under the receiver operating characteristic curve [AUC] 0.759, 95% CI 0.595–0.924) (Lang et al., 2017). In a separate cohort of 57 patients with pathology-confirmed pulmonary TB, including 30 with symptomatic and 27 with asymptomatic disease, 25 cases (44%) were misclassified as lung cancer on PET/CT (Yu et al., 2019).

Substantial bacillary burden may also coexist with a normal CXR. During a Malaysian outbreak, 42.9% of smear-positive cases had normal radiographs, and asymptomatic individuals had higher odds of this presentation, although the estimate was imprecise (crude odds ratio 12.0, 95% CI 1.12–128.84) (Mohd Redhuan et al., 2024). Imaging also showed limited discrimination by culture status: culture-negative subclinical pulmonary TB was less often bilateral than culture-positive disease on both CXR (8.5% versus 32.0%) and CT (15.4% versus 42.9%), but no other radiographic pattern reliably distinguished the groups (Chansaengpetch et al., 2024).

Thus, CXR is valuable for screening and triage but cannot independently determine disease activity or exclude early, paucibacillary, or anatomically subtle TB. Individuals at high epidemiological risk, particularly those with discordant clinical, microbiological, and radiographic findings, may require enhanced microbiological testing, CT, or longitudinal reassessment.

### Tension between screening strategy and resource allocation

Diagnosing asymptomatic TB is as much a challenge of programme design as of test performance. Because symptom-negative individuals rarely seek care, detection requires proactive, often symptom-independent screening of selected high-risk populations. However, broader screening increases demands for imaging, specimen collection, molecular testing, referral and follow-up, while generating more false-positive results and uncertain treatment decisions. These trade-offs favor context-specific, risk-stratified screening strategies rather than indiscriminate expansion of testing (World Health Organization, 2022).

Disease may also be missed when evaluation is restricted to the clinically affected organ. Among 68 patients initially classified as having bone TB alone, 44 underwent imaging that included the entire lung fields; 41 (93.2%) had pulmonary parenchymal abnormalities, including 35 (79.5%) with only minimal changes suggestive of subclinical pulmonary TB (Yin et al., 2024). Genomic and epidemiological analyses suggested, but did not establish, transmission from patients with unrecognized pulmonary involvement (Yin et al., 2024). Similarly, urine culture or molecular testing identified clinically silent urogenital TB in 7 of 102 patients with pulmonary TB; four had no corresponding abnormality on imaging or endoscopy (Zachoval et al., 2018).

Conventional risk-stratification tools may also be insufficient. Among 525 household contacts in India, 29 (5.5%) were culture-positive, of whom 28 were asymptomatic and only two had CXR findings suggestive of TB. The Infectivity Score showed limited discrimination for identifying subclinical TB (AUC 0.61, 95% CI 0.52–0.70) (Bekken et al., 2020). Among people who inject drugs in Vietnam, computer-aided detection (CAD)-assisted CXR followed by Xpert testing increased sensitivity from 43.9% to 82.9% and diagnostic yield from 1.7% to 3.2%, but reduced positive predictive value to 54.0% and increased the cost per case detected from US$309 to US$439 (D'Ottavi et al., 2025). Screening strategies should therefore be evaluated by diagnostic yield, confirmatory capacity, false-positive referrals, linkage to care, treatment uptake and opportunity costs.

## Emerging tools and multimodal strategies

### Host biomarkers

Host biomarkers, including transcriptomic signatures, circulating proteins, and inflammatory and metabolic markers, are attractive because they do not require sputum and could support early detection, triage and risk stratification (Greenan-Barrett et al., 2025; Pedersen et al., 2025; Sun et al., 2025). However, host responses in asymptomatic TB are generally weaker and more heterogeneous than those in symptomatic disease, and most candidates remain investigational (Awany et al., 2026; Mendelsohn et al., 2025; Zhang et al., 2025).

In an individual-participant-data analysis of 6,530 samples from 5,185 individuals, five single-gene transcripts (*BATF2*, *FCGR1A/B*, *ANKRD22*, *GBP2* and *SERPING1*) performed similarly to the Roe3 multigene signature in distinguishing subclinical TB from individuals who remained disease-free for 12 months, with AUCs of 0.75–0.77. None met the WHO minimum target product profile for a TB progression test (Greenan-Barrett et al., 2025). Of 283 samples classified as subclinical TB, only 39 represented prevalent asymptomatic disease; the remaining 244 were obtained from individuals who subsequently developed incident TB and were therefore presumed to have had subclinical disease at sampling. Performance declined with increasing time to diagnosis (Greenan-Barrett et al., 2025). An 11-gene signature (*ABLIM2*, *C20orf197*, *CTC-543D15.3*, *CTD-2503O16.3*, *HLA-DRB3*, *METRNL*, *RAB11B-AS1*, *RP4-614C10.2*, *RNA5SP345*, *RSU1P1* and *UACA*) also differentiated subclinical from incipient TB in an Indian household-contact cohort, but was derived from only 12 and 35 cases, respectively, raising concerns about overfitting (Sivakumaran et al., 2023).

South African transcriptomic and proteomic profiling showed that asymptomatic and symptomatic TB shared innate, interferon-associated and inflammatory responses, although these were attenuated in asymptomatic disease. Integrated analyses identified two stable molecular clusters of asymptomatic TB that differed in bacillary burden, inflammatory activity and radiographic severity. A three-gene signature (*PSTPIP2*, *CARD16* and *LIMK1*) achieved AUCs of 0.833 in the discovery set and 0.839 in the hold-out set, but did not significantly outperform published signatures in hold-out or external validation (Awany et al., 2026).

Simpler biomarkers derived from routine complete blood counts, including the monocyte-to-lymphocyte ratio (MLR) and neutrophil-to-lymphocyte ratio (NLR), have also been evaluated as low-cost markers of TB. A systematic review of 41 studies involving 11,952 participants found that higher MLR and NLR were associated with TB disease compared with healthy individuals or those with TB infection; however, thresholds varied substantially across studies, precluding robust pooled estimates of diagnostic accuracy (Fritschi et al., 2023). In a separate clinical evaluation, MLR showed only modest discriminatory performance, with sensitivities of 58.0–62.5% and specificities of 50.0–70.0%, and was influenced by sex and disease severity (Buttle et al., 2021). Evidence specifically addressing MLR or NLR for detection of asymptomatic TB remains limited. These indices may therefore have value as inexpensive adjunctive markers, but their current performance and lack of standardized thresholds limit their use as standalone screening tools for asymptomatic TB.

Among South African household contacts, serum C-reactive protein poorly distinguished asymptomatic TB from healthy contacts (AUC 0.60, 95% CI 0.47–0.73) (Mendelsohn et al., 2025), consistent with the attenuated inflammatory profiles reported in subclinical disease (Min et al., 2020; Tang et al., 2022; Zhang et al., 2025). Extracellular newly identified receptor for advanced glycation end products-binding protein (EN-RAGE) combined with monocyte chemoattractant protein 3 (MCP-3) achieved AUCs of 0.90 in an Olink discovery cohort and 0.837 in an independent enzyme-linked immunosorbent assay cohort for distinguishing asymptomatic TB from healthy controls and individuals with latent TB infection. However, both proteins were also elevated in non-TB pulmonary diseases, limiting disease specificity (Sun et al., 2025). In a Vietnamese active case-finding cohort, TB disease was defined by a positive GeneXpert result together with either positive sputum culture or chest radiography consistent with active TB. Against this case definition, plasma interferon-γ-induced protein 10 achieved an AUC of 0.708, with 95.5% specificity but 32.9% sensitivity. A nine-protein panel increased the AUC to 0.739, but sensitivity remained low at 36.5% (Pedersen et al., 2025).

### CAD, PET/CT, and AI-assisted screening

CAD and AI-assisted CXR are well suited to large-scale screening because they increase throughput and standardize image interpretation, but they remain triage tools rather than substitutes for microbiological confirmation. In a mobile screening programme in Nigeria, approximately 66% (115/174) of pulmonary TB cases detected by GeneXpert did not have chronic cough. Follow-up of individuals with CAD4TB scores ≥80 despite an initially negative GeneXpert result from a single spot-sputum specimen identified 11 additional cases (Eneogu et al., 2024).

CAD thresholds must be calibrated to the target population, disease prevalence, confirmatory-testing capacity and available resources. Lower thresholds increase sensitivity but generate more false-positive referrals, whereas higher thresholds reduce laboratory demand at the cost of missed disease. In a pooled analysis of five community-based active case-finding studies in South Africa, prevalent TB was microbiologically confirmed by Xpert MTB/RIF and/or culture using routine or enhanced sputum sampling, whereas incident TB was identified during longitudinal follow-up. CAD4TB v7 achieved an overall AUC of 0.83 but performed less well in asymptomatic than symptomatic individuals (0.79 versus 0.85) and did not meet the WHO target product profile for a TB screening or triage test (Scott et al., 2025).

At 90% sensitivity, specificity was only 44.9% (Scott et al., 2025). CAD also cannot fully overcome the limitations of radiographic triage. In rural South Africa, a radiologist classified 19 of 99 microbiologically confirmed cases (19.2%) as having normal lung fields. Fifteen were asymptomatic and would have been missed by symptom- and radiologist-based triage. Detection required a low CAD4TB v5 threshold of 25, which referred 59.6% of participants for sputum testing (Fehr et al., 2021). Thus, CAD can improve detection beyond symptom-based screening, but its yield and efficiency depend on threshold selection, population characteristics, confirmatory-sampling intensity and management of the diagnostic cascade.

PET/CT is unsuitable for routine screening but provides a sensitive imaging benchmark for characterizing asymptomatic TB. Among 250 initially asymptomatic, HIV-negative household contacts of patients with rifampicin-resistant TB, 11 of 16 participants with bacteriologically confirmed TB (69%) remained asymptomatic at confirmation, and 10 of these 11 (91%) had baseline PET/CT findings consistent with TB (Esmail et al., 2026). Operational studies further illustrate both the potential and limitations of AI-assisted screening. In predominantly tribal regions of India, 20 of 51 asymptomatic individuals flagged by AI were diagnosed with TB using a composite microbiological or clinical reference standard (39.2%, 95% CI 25.8–53.9) (Gupta et al., 2026). Among participants diagnosed with TB, AI scores were similar in asymptomatic and symptomatic individuals.

### Multimodal algorithms

Future diagnostic strategies will probably rely on staged, multimodal algorithms rather than any single test. Risk assessment and CAD-assisted CXR could determine who undergoes microbiological testing, with culture, non-sputum assays, enhanced sampling and follow-up reserved for negative or discordant results. Among household contacts of patients with rifampicin-resistant TB, routine testing of a single spontaneously produced sputum sample by Xpert MTB/RIF detected only 7 of 23 prevalent cases. Of those exceeding commonly used or manufacturer-recommended CAD thresholds but not diagnosed by routine single-sputum testing, 30–35% were subsequently diagnosed through enhanced baseline sputum investigation or follow-up and should therefore not automatically be considered to have false-positive CAD results (Macpherson et al., 2025). Escalation may include repeated or induced sputum collection, culture, alternative specimens or reassessment, guided by radiographic severity and risk. However, additional tests should be adopted only when they provide incremental value: in asymptomatic, HIV-negative participants, blood biomarkers did not materially improve CAD performance (Macpherson et al., 2025). Effective algorithms therefore require explicit escalation, referral, follow-up, and exit criteria, rather than simply combining every available test.

## Discussion

Asymptomatic tuberculosis is not a uniform or benign state, but a symptom-defined phenotype within a dynamic disease spectrum. The absence of reported symptoms should therefore not be interpreted as evidence of microbiological inactivity, radiographic normality, or negligible risk of progression or transmission. Its heterogeneous presentation—ranging from minimal or intermittently detectable disease to radiographically advanced and microbiologically positive tuberculosis—explains why symptom-based screening alone is insufficient.

No single diagnostic approach can reliably identify or exclude asymptomatic tuberculosis. Sputum-based tests are constrained by specimen availability and reduced sensitivity in paucibacillary disease, whereas chest radiography and computer-aided detection provide scalable triage but lack sufficient specificity and confirmatory value. Host biomarkers and non-sputum assays are promising, but most remain insufficiently validated for routine clinical or programmatic use. The most defensible strategy is therefore not to seek a standalone test, but to develop staged, risk-stratified diagnostic pathways that integrate epidemiological risk, imaging, microbiological confirmation, enhanced specimen collection and longitudinal reassessment.

Such pathways should also account for age-specific diagnostic challenges. Performance estimates derived predominantly from adult populations cannot be assumed to apply directly to children, in whom symptom assessment, specimen acquisition, microbiological confirmation and radiographic interpretation differ substantially. In addition, as this narrative Review was informed by a targeted, non-systematic PubMed search, relevant studies may have been missed and the evidence synthesized here is susceptible to selection bias. Future research should therefore move beyond isolated measures of diagnostic accuracy and evaluate complete diagnostic and management pathways in the populations and settings in which they are intended to be implemented (**Table 1**).

**Table 1:** Future research priorities for asymptomatic tuberculosis.

Priority area	Key research direction	Why it matters
1. Standardize symptom ascertainment	Establish standardized definitions and methods for symptom ascertainment, including the symptom domains assessed, recall periods, severity thresholds, questioning approaches and timing relative to diagnostic evaluation.	Consistent ascertainment is essential for comparing findings across studies and reducing misclassification of asymptomatic tuberculosis.
2. Define the natural history of asymptomatic TB	Conduct prospective longitudinal studies using serial symptom assessment, microbiological testing, imaging and biomarkers to characterize resolution, persistence, fluctuation, progression and recurrence across distinct asymptomatic tuberculosis phenotypes.	The natural history of asymptomatic tuberculosis remains poorly defined, limiting prognostic stratification and evidence-based management.
3. Measure infectiousness directly	Measure infectiousness directly by integrating bacillary burden, aerosol generation, respiratory activity, exposure conditions, molecular epidemiology and outcomes among contacts, rather than inferring transmission potential from symptoms alone.	Reliable estimates of transmission risk are needed to guide infection-control measures, contact investigation and public health interventions.
4. Risk-stratify active case finding	Develop and validate risk-stratified active case-finding strategies for populations and settings in which screening is most likely to provide clinical and public health benefit.	Targeted approaches are more likely than untargeted screening to achieve an acceptable balance among diagnostic yield, feasibility, resource use and clinical impact.
5. Evaluate diagnostics as care pathways	Assess diagnostic strategies as end-to-end care pathways encompassing triage, specimen acquisition, confirmatory testing, management of discordant findings, referral, treatment initiation, longitudinal reassessment and exit criteria. Evaluation should include diagnostic yield, false-positive referrals, linkage to care, patient outcomes and resource requirements.	Strong analytical performance of an individual test may not translate into clinical or programmatic benefit when specimen collection, confirmation, referral or follow-up is inadequate.
6. Determine optimal treatment strategies	Determine which phenotypes benefit from preventive treatment, standard multidrug therapy, modified regimens or structured surveillance within research settings, according to biological activity, bacillary burden, diagnostic certainty and risks of progression and transmission.	Management should balance treatment toxicity, overtreatment and patient burden against the potential benefits of reducing disease progression, recurrence, transmission and post-tuberculosis lung disease.

## Conclusions

Asymptomatic TB represents a heterogeneous, symptom-defined phenotype spanning a broad range of microbiological and radiographic manifestations. Its detection is limited by imperfect symptom ascertainment, difficulty obtaining respiratory specimens and variable bacillary burden, while imaging, non-sputum assays and host biomarkers provide complementary but currently insufficient evidence for diagnostic confirmation when used alone. Effective diagnostic strategies will therefore require staged, risk-stratified approaches that combine epidemiological risk assessment, imaging, microbiological testing, enhanced specimen collection and longitudinal reassessment.

Key uncertainties remain regarding the natural history and infectiousness of asymptomatic TB, the optimal definitions for research and clinical practice, the performance of emerging diagnostics in children and other underrepresented populations, and the circumstances in which treatment should be favored over structured surveillance. Addressing these gaps will require prospective longitudinal studies and evaluation of complete diagnostic and management pathways rather than isolated assessments of test performance.
